# Supplementary material for: Predictors of natively unfolded proteins: unanimous consensus score to detect a twilight zone between order and disorder in generic datasets
Source: BMC Bioinformatics. 2010 Apr 21;11:198. doi: 10.1186/1471-2105-11-198 (PMC2877690; doi:10.1186/1471-2105-11-198)
Supplement: Additional file 1 — Supplemental table ST1. This file contains supplemental Table ST1, with caption. [file 1471-2105-11-198-S1.DOC]

## Supplemental table ST1 - Performance of single folding indexes for dataset C purged from complexed proteins

|  | *S_n_* | *S_p_* | *f_p_* | nc |
| --- | --- | --- | --- | --- |
| Poodle-W | 0.75 | 0.86 | 0.14 | 0 |
| *gVSL2* | 0.82 | 0.75 | 0.25 | 0 |
| *<E_c_>* | 0.74 | 0.81 | 0.19 | 0 |
| *<P>* | 0.72 | 0.80 | 0.20 | 0 |
| *HQ* | 0.69 | 0.74 | 0.26 | 0 |
| *S_U_* | 0.63 | 0.94 | 0.06 | 0 |
| *S_V_* | 0.86 | 0.66 | 0.34 | 0 |
| *S_SU_* | 0.82 | 0.92 | 0.08 | 0.28 |

Performance of *HQ*, mean packing *<P>*, mean pairwise energy *<E_c_>*, *gVSL2,* Poodle-W, *S_U_*, *S_V_* and *S_SU_* in discriminating natively unfolded proteins from folded ones among those in our own set C purged of the 506 complexed proteins. Mean packing threshold is fixed at 20.55 and mean pairwise energy threshold is fixed at -0.37 a.e.u. *nc* is the fraction of unclassified proteins
