# Supplementary material for: Predictors of natively unfolded proteins: unanimous consensus score to detect a twilight zone between order and disorder in generic datasets
Source: BMC Bioinformatics. 2010 Apr 21;11:198. doi: 10.1186/1471-2105-11-198 (PMC2877690; doi:10.1186/1471-2105-11-198)
Supplement: Additional file 2 — Supplemental figure S1. This file contains supplemental figure S1, with caption. [file 1471-2105-11-198-S2.DOC]

##
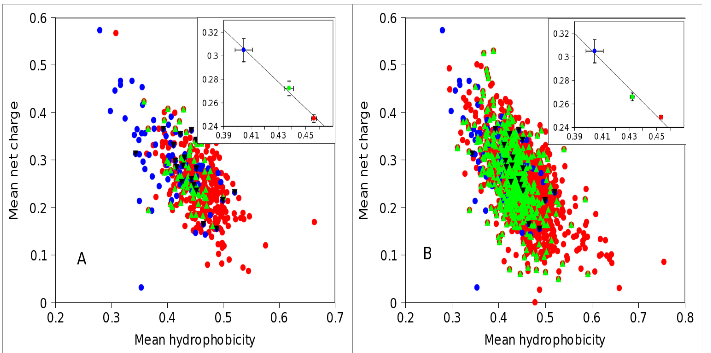


## Figure S1. Hydrophobicity/charge plot of folded, unfolded and unclassified proteins in set C

Hydrophobicity/charge plots of proteins in set C. Red dots refer to experimentally folded, blue dots to experimentally unfolded proteins; whereas upper green triangles refer to experimentally folded proteins unclassified by *S_SU_* and lower black triangles to experimentally unfolded proteins unclassified by *S_SU_*. **1A.** Proteins in set C, purged by folded complexes and ligand binding proteins. **1B**. Proteins in set C, purged by those experimentally folded proteins that are not complexes and ligand binding proteins. Therefore the red dots and upper green triangles in figure 1B refer to those proteins that have been excluded from 1A. Note, in figure 1A, the narrowing of the overlap area with respect to the case of the entire set C (figure 3 of the paper). It is remarkable that the centroids of the unclassified proteins are always intermediate both in the whole set C (figure 3), in the set purged by untypical proteins (supplementary figure 1A) and in its complement (supplementary figure 1B).
