# Supplementary material for: Predictors of natively unfolded proteins: unanimous consensus score to detect a twilight zone between order and disorder in generic datasets
Source: BMC Bioinformatics. 2010 Apr 21;11:198. doi: 10.1186/1471-2105-11-198 (PMC2877690; doi:10.1186/1471-2105-11-198)
Supplement: Additional file 3 — Supplemental figure S2. This file contains supplemental figure S2, with caption. [file 1471-2105-11-198-S3.DOC]

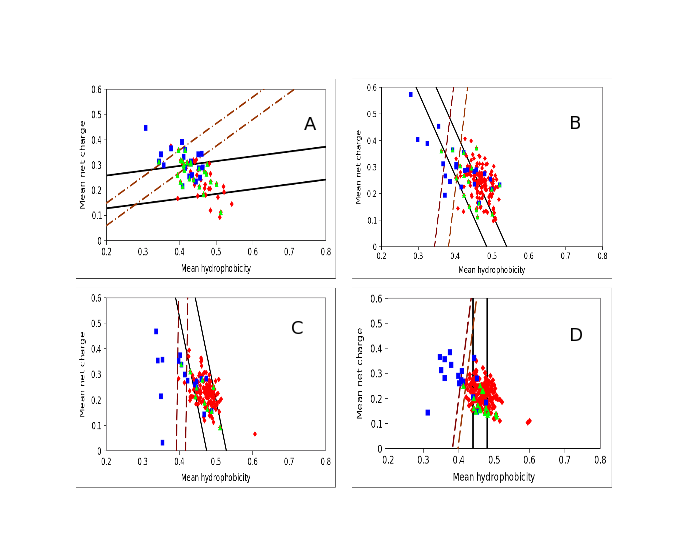


**Figure S2. Extension of the projections of the twilight zone in the hydrophobicity/charge plane for different chain length bins**

Extension of the twilight zone in the hydrophobicity/charge plane evaluated in four different chain length bins for proteins in set B: a) proteins with length between 50 and 99 amino acids; b) proteins with length between 100 and 199 amino acids; c) proteins with length between 200 and 299 amino acids; d) proteins longer than 300 amino acids. Solid lines delimit the twilight zone as selected by the method described in the Methods section of the paper; dashed lines refer to a twilight zone determined by a logistic regression similar to that in ref. [24]. Note the narrowing of the bands as chain length increases. Blue squares: experimentally determined unfolded proteins; red diamonds: experimentally determined folded proteins. Green triangles refer to those proteins which *S_SU_* does not classify.
