# Supplementary material for: Predictors of natively unfolded proteins: unanimous consensus score to detect a twilight zone between order and disorder in generic datasets
Source: BMC Bioinformatics. 2010 Apr 21;11:198. doi: 10.1186/1471-2105-11-198 (PMC2877690; doi:10.1186/1471-2105-11-198)
Supplement: Additional file 4 — PDB entries of folded proteins in set C. This file collects the PDB entries of the 2369 proteins in set C. [file 1471-2105-11-198-S4.DOC]

**PDB entries of folded proteins in set C**

1a23_

1a56_

1a63_

1a6x_

1a7i_

1a90_

1a93A

1aab_

1abv_

1adx_

1aey_

1afoA

1afp_

1ag2_

1ag4_

1ah9_

1aiw_

1aj4_

1aje_

1ajyA

1akp_

1aoy_

1ap7_

1apf_

1apq_

1aps_

1aq5A

1aqa_

1arqA

1aty_

1auuA

1aw0_

1ax3_

1ayj_

1az6_

1b1a_

1b2tA

1b8qA

1b9pA

1b9uA

1ba4_

1bak_

1bax_

1bb8_

1bby_

1bc4_

1bc9_

1bci_

1bf9_

1bgk_

1bh7_

1bhi_

1bj8_

1bjx_

1bku_

1bl1_

1bm4A

1bnb_

1bnxA

1bo9A

1boeA

1bor_

1bpv_

1br0A

1brz_

1bshA

1bujA

1buyA

1bv8A

1bw6A

1bxdA

1by1A

1by6A

1bymA

1bzg_

1c01A

1c05A

1c07A

1c17M

1c3yA

1c53_

1c5a_

1c6wA

1ccvA

1ce4A

1cejA

1ceuA

1cf4B

1cfh_

1chvS

1ci5A

1cirA

1cjgA

1ck2A

1ckv_

1cl3A

1clh_

1cmr_

1co0A

1co4A

1cokA

1copD

1couA

1cpzA

1cwxA

1cx1A

1cxwA

1cyl_

1cz4A

1d0rA

1d1dA

1d1nA

1d4bA

1d4uA

1d6gA

1d8bA

1d8jA

1d9aA

1daqA

1dcjA

1ddbA

1ddmA

1de1A

1dec_

1dg4A

1dguA

1dgzA

1dipA

1dk2A

1dkcA

1dl0A

1dmc_

1dnyA

1dp3A

1dqcA

1dro_

1dsvA

1du6A

1dujA

1dv0A

1dv9A

1dxzA

1dz1A

1e0eA

1e0gA

1e2b_

1e3yA

1e4uA

1e52A

1e5bA

1e5uI

1e7jA

1e8eA

1e8pA

1e9kA

1eal_

1eciB

1ed7A

1edsA

1edvA

1edxA

1ef4A

1ef5A

1ego_

1egxA

1ehs_

1ei0A

1eigA

1eikA

1eit_

1ekzA

1emwA

1erd_

1ev0A

1ewiA

1ewsA

1exeA

1exg_

1exkA

1eyfA

1eza_

1ezeA

1eztA

1f0zA

1f40A

1f4sP

1f53A

1f6gA

1f81A

1fafA

1faq_

1fct_

1fdm_

1fexA

1fh1A

1fjcA

1fjkA

1fliA

1fmhA

1fo5A

1fow_

1fsb_

1fshA

1ftt_

1fu5A

1fwoA

1fwp_

1fwqA

1fyjA

1fztA

1g10A

1g1zA

1g25A

1g3gA

1g47A

1g4fA

1g5mA

1g5vA

1g6eA

1g6zA

1g7eA

1g84A

1g90A

1ga3A

1gccA

1gcf_

1gdc_

1gdf_

1ge9A

1gh1A

1gh8A

1gh9A

1ghhA

1ghj_

1gjxA

1gks_

1gl5A

1gnc_

1gp8A

1gps_

1gur_

1gw3_

1gxeA

1gxgA

1gya_

1h0tB

1h1jS

1h3zA

1h5pA

1h67A

1h6qA

1h8bA

1h8cA

1h9eA

1ha8A

1hae_

1hcd_

1hd6A

1hdlA

1hdp_

1hf9A

1hfh_

1hhnA

1hi7A

1hic_

1hj0A

1hk6A

1hks_

1hkyA

1hllA

1hn3A

1hn6A

1hnr_

1hp3A

1hpwA

1hqbA

1hryA

1hs5A

1hs7A

1hv2A

1hx2A

1hy9A

1i17A

1i18A

1i1sA

1i26A

1i35A

1i4vA

1i5hW

1i6zA

1ib8A

1ib9A

1iba_

1ica_

1ifyA

1ig4A

1igl_

1ihvA

1iieA

1iijA

1iioA

1ikl_

1iltA

1ilyA

1imoA

1imt_

1imuA

1iouA

1ioxA

1ip9A

1irf_

1irl_

1irsA

1irzA

1iurA

1iuyA

1ivtA

1ivzA

1iw4A

1iwcA

1iycA

1iyrA

1j0gA

1j0sA

1j0tA

1j26A

1j2mA

1j3gA

1j4wA

1j6qA

1j6yA

1j7qA

1j9iA

1jbaA

1jbiA

1jdmA

1jeiA

1jfwA

1jjdA

1jjrA

1jkzA

1jli_

1jm7A

1jn7A

1jo5A

1jo6A

1joyA

1jqsB

1jqsC

1jr5A

1jrmA

1jruA

1jt8A

1junA

1jvr_

1jw2A

1jw3A

1jxcA

1jzuA

1k0hA

1k0pA

1k1gA

1k1vA

1k2mA

1k36A

1k3kA

1k42A

1k4uP

1k81A

1k8bA

1k8hA

1k8vA

1k99A

1kbfA

1kbhA

1kd6A

1kdxA

1kftA

1kg1A

1kj5A

1kjkA

1klaA

1klrA

1klvA

1kn6A

1ksr_

1ktuA

1kul_

1kv4A

1l1pA

1l3eA

1l3gA

1l3hA

1l3oA

1l3yA

1l8cA

1lknA

1ll8A

1lmmA

1lpvA

1lr1A

1lupA

1lv3A

1lv9A

1ly7A

1lyp_

1m0vA

1m2eA

1m2sA

1m5zA

1m7lA

1m94A

1m9oA

1mkcA

1mknA

1mm0A

1mm4A

1mp1A

1mr4A

1mszA

1muzA

1mw4A

1mzkA

1n0zA

1n6zA

1nblA

1ncs_

1nct_

1nd9A

1neiA

1neq_

1nglA

1njqA

1nr3A

1nre_

1ns1A

1nsoA

1nvoA

1nwbA

1nxiA

1ny4A

1ny8A

1ny9A

1nz9A

1o0pA

1o7bT

1o8rA

1oh1A

1omb_

1oo9B

1op4A

1ot4A

1otrA

1ovqA

1ow5A

1oy2A

1oyiA

1p0aA

1p1dA

1p4qA

1p4wA

1p7mA

1p8bA

1p94A

1p97A

1p9cA

1p9kA

1paa_

1pavA

1pb5A

1pba_

1pbuA

1pc2A

1pce_

1pd6A

1pd7A

1pdc_

1peh_

1pfjA

1pfsA

1pft_

1pgyA

1pih_

1pls_

1pn5A

1pnbA

1pou_

1pqsA

1pqxA

1pra_

1psyA

1pulA

1puzA

1pv0A

1pveA

1pvzA

1pxeA

1pxqA

1pyc_

1pyvA

1pzqA

1pzrA

1q01A

1q02A

1q1vA

1q2iA

1q2zA

1q3tA

1q3yA

1q53A

1q5wA

1q60A

1q68A

1q80A

1q9fA

1qa4A

1qckA

1qeyA

1qfqB

1qgmA

1qhkA

1qkfA

1qldA

1qloA

1qp2A

1qp6A

1qpmA

1qqiA

1r21A

1r48A

1r4gA

1r4kA

1r4tA

1r5eA

1r5sA

1r79A

1r7gA

1r9pA

1rduA

1res_

1rfa_

1rfhA

1ri0A

1ri9A

1rimA

1rklA

1rl1A

1rmkA

1roo_

1rq6A

1rqsA

1rqtA

1rrzA

1rsoA

1rsoB

1rw5A

1rykA

1rzsA

1s04A

1s3aA

1s4zC

1s62A

1s6dA

1s6xA

1s7aA

1s7eA

1s8kA

1sb6A

1sboA

1sf0A

1sg5A

1sg7A

1sgoA

1sis_

1sjqA

1skhA

1snlA

1so9A

1spf_

1sqrA

1sr2A

1srkA

1sro_

1ss3A

1sseA

1sskA

1sslA

1ssn_

1suyC

1szvA

1t0cA

1t0vA

1t17A

1t1hA

1t23A

1t3kA

1t3oA

1t4lB

1t50A

1tam_

1tbaA

1tbaB

1tbd_

1te7A

1tf3A

1tfi_

1th5A

1tknA

1tlhB

1tm9A

1tmzA

1totA

1tpn_

1tq1A

1trlA

1ttnA

1tu2B

1tujA

1tuzA

1tviA

1tvjA

1tvs_

1tykA

1u34A

1u3nA

1u3oA

1u57A

1u5mA

1u5sA

1u81A

1u89A

1uapA

1ucpA

1ucvA

1ue9A

1ueoA

1uepA

1ufmA

1ufwA

1ufzA

1ug1A

1ug2A

1ug7A

1ug8A

1uglA

1uhcA

1uhmA

1uhsA

1uhwA

1uilA

1ujlA

1ujrA

1ujtA

1ujvA

1uk5A

1ukxA

1ul7A

1upnB

1upnC

1upnD

1uqvA

1urfA

1utaA

1uvfA

1uw0A

1uw2A

1uzcA

1v1cA

1v1dA

1v2yA

1v31A

1v32A

1v38A

1v5mA

1v61A

1v64A

1v65A

1v85A

1v88A

1v92A

1v9vA

1vazA

1vcsA

1vd4A

1veeA

1vehA

1vejA

1vekA

1vib_

1vig_

1vkrA

1vna_

1vryA

1vtx_

1vyxA

1vzsA

1w09A

1w1nA

1w2qA

1w3dA

1wa8A

1wa8B

1wazA

1wcnA

1wd2A

1we7A

1we9A

1welA

1wexA

1wf5A

1wf6A

1wffA

1wfkA

1wfmA

1wfqA

1wg1A

1wg7A

1wgdA

1wgeA

1wgnA

1wgpA

1wgsA

1wguA

1wgwA

1wgxA

1wgyA

1wh5A

1wh9A

1whlA

1whqA

1whrA

1wi3A

1wi5A

1wi9A

1wicA

1widA

1wigA

1wiiA

1wijA

1wikA

1wilA

1wimA

1wixA

1wj0A

1wj7A

1wjjA

1wjqA

1wjtA

1wjvA

1wkiA

1wkt_

1wloA

1wlxA

1wpiA

1wqbA

1wrfA

1wsoA

1wsxA

1wt8A

1wu0A

1wuzA

1wvzA

1wwgA

1wwqA

1wwuA

1wx7A

1wxnA

1wxpA

1wxuA

1x32A

1x3aA

1x3cA

1x3uA

1x43A

1x49A

1x4eA

1x4vA

1x4yA

1x58A

1x62A

1x63A

1x67A

1x6gA

1x93A

1x9aA

1x9vA

1xc0A

1xc5A

1xhhA

1xi7A

1xkeA

1xm0A

1xn5A

1xn7A

1xn8A

1xoyA

1xpnA

1xpvA

1xr0B

1xrdA

1xs3A

1xs9A

1xu6A

1y0jB

1y4oA

1y5oA

1y6uA

1y74A

1y74B

1y76A

1y7kA

1y7qA

1y7xA

1y9xA

1ybjA

1yelA

1yezA

1yfbA

1ygmA

1yhdA

1yhpA

1ykgA

1ynxA

1yo4A

1ys5A

1yua_

1yub_

1yuiA

1ywlA

1yx0A

1yzsA

1z00A

1z00B

1z1dA

1z1zA

1z2gA

1z3rA

1z65A

1z8mA

1z8rA

1z8sA

1z99A

1z9eA

1z9iA

1z9vA

1zaeA

1zaq_

1zc1A

1zdvA

1zfuA

1zguA

1zn5A

1zr9A

1zriA

1zrrA

1zrxA

1ztn_

1zu1A

1zufA

1zw8A

1zwtA

1zxfA

1zyiA

1zzpA

2a02A

2a05A

2a2vA

2a3jA

2a4hA

2a7oA

2a7yA

2ab9A

2acmA

2acmB

2adzA

2afdA

2afjA

2aghC

2ai6A

2aivA

2aizP

2ajeA

2akkA

2al3A

2aqeA

2arfA

2ariA

2aswA

2asyA

2auvA

2ayyA

2b0gA

2b0hA

2b19A

2b1wA

2b5bA

2b5xA

2b68A

2b7eA

2b9kA

2b9zA

2ba3A

2baiA

2bds_

2bgoA

2bi6H

2bicA

2bn5A

2bn8A

2bttA

2bvbA

2byeA

2bz2A

2bztA

2c5zA

2c6aA

2c7hA

2ch0A

2ck5A

2ckaA

2cknA

2cocA

2codA

2cpiA

2cpxA

2cpyA

2cq8A

2cqjA

2cqoA

2criA

2crjA

2crqA

2cskA

2csvA

2cswA

2ct0A

2ct5A

2ctlA

2cuhA

2cw1A

2czyA

2d3jA

2d46A

2d56A

2d7nA

2d8bA

2d8cA

2d8mA

2d9gA

2d9kA

2da6A

2da7A

2daeA

2dafA

2dajA

2dalA

2davA

2dazA

2db2A

2dbjA

2dcoA

2dcwA

2ddlA

2dgvA

2digA

2diiA

2djjA

2djmA

2dk5A

2dk6A

2dklA

2dkyA

2dleA

2dmeA

2dmiA

2dmjA

2dogA

2dq5A

2dt6A

2dt7A

2dw3A

2dy7A

2dy8A

2dzlA

2e0gA

2e2wA

2e30B

2e5rA

2e5tA

2e60A

2e61A

2e62A

2e6iA

2e6oA

2e6qA

2e6zA

2e70A

2e72A

2e7gA

2e8pA

2eriA

2ersA

2esyA

2ev8A

2ezh_

2ezk_

2ezwA

2f09A

2f3iA

2f40A

2f8bA

2f93B

2fc6A

2fc7A

2fcdA

2fejA

2fftA

2ffwA

2fgxA

2fjlA

2fkxA

2fmcA

2fmr_

2fn5A

2fqhA

2fs1A

2fv4B

2fwlB

2fwsA

2fwuA

2fxpA

2fz0A

2g0uA

2g2bA

2g31A

2g35A

2g3qA

2g7jA

2gb8A

2gcxA

2gdlA

2ge9A

2ghfA

2gi4A

2gj0A

2gjiA

2gjyA

2gloA

2glwA

2gm2A

2gmgA

2gmoA

2govA

2gqbA

2gqeA

2gqkA

2gtvX

2gw6A

2gzuA

2h1zA

2h35A

2h3aA

2h41A

2h7eB

2h8bB

2hacA

2hbpA

2hc5A

2hdlA

2hdpA

2hepA

2hfdA

2hfiA

2hfqA

2hg7A

2hgcA

2hgf_

2hh8A

2hhiA

2hj8A

2hjjA

2hm6A

2hn8A

2hnaA

2hpuA

2hqoA

2hwtA

2hx6A

2hzdA

2i3bA

2i5oA

2i68A

2i8lA

2i9sA

2i9yA

2ic4A

2idaA

2ifo_

2igrA

2ihxA

2iueA

2ivwA

2j2sA

2j5dA

2j5hA

2j5oA

2j8pA

2jm1A

2jm2A

2jm3A

2jmoA

2jmpA

2jmvA

2jmxA

2jn4A

2jn9A

2jneA

2jnjA

2jo8A

2jodA

2jodB

2jouA

2jovA

2jp2A

2jp7A

2jpeA

2jq4A

2jq5A

2jqfR

2jqqA

2jrbA

2jrjA

2jrmA

2jrrA

2jrzA

2ncm_

2nef_

2oddA

2odxA

2ofqA

2oqpA

2outA

2pldA

2pta_

2sh1_

2u2fA

2uwqA

2vgh_

2vik_

3bbg_

3leu_

3mspA

3ncmA

4ull_

4znf_

7gatA

1txyB

1m5iA

1l2wI

2dr3A

1ic2B

1y0nA

2dreC

2oqqA

1pzwA

1r5qA

1sfuA

1q2yA

1ye5A

1mbyA

1gd2I

1znoA

2c3gA

2iyjA

1dtjB

2hp7A

2j9uB

1aly_

1aol_

2g6qA

2h00A

2qbvA

1inr_

2fhjA

2hdzA

1y96B

2i9fC

1fbtA

1ty2C

2czvC

2dobA

1gl4B

2h3gX

1wq6A

2a8iA

1oa9A

1d3bA

1d3bB

1vkwA

1dqtA

2oq0A

1mi8A

2hqlA

1pn9A

1t5rB

1el6A

1mzgB

1n2dC

2guzB

2uwjE

2uwjF

1tig_

1ygaA

1i7wD

1rfeA

2huoA

1lddA

1pi1A

1yd6A

2ib0A

1bysA

1h6kZ

1h8eI

2ervA

2v3kA

1fc3A

1hbkA

1tafA

2ppxA

1dvoA

2h8lA

4mt2_

2bkqC

2fxfA

1mzwB

2g7cA

2hq7A

1poc_

1rljA

2d28C

2f4wA

1x6jA

1zljD

1sn8A

2bw3B

1mkbA

2fnoA

1vjnA

1rpkA

2ga1A

2i9cA

2fw5A

2ifxA

1u7eB

1nkzA

1tl2A

1clvI

2j83A

1yzvA

2c21A

1uz0A

2amuA

1wmyA

2aoaB

2gy9K

2gy9T

2gyaX

2gybN

2gybQ

2gyc1

1l3pA

2d0wA

2dyoB

1rhfA

1lg7A

2j6zA

2pkhA

1y71B

2f6qA

2j7zA

2nzcD

2o4tA

2apoA

2ouwA

1ptq_

1bea_

1zdeA

1yliA

2z20A

1y12C

2g7fA

1hleA

1pb0A

1o4tA

1vchE

1zv8A

2c49A

2pfrB

2e7vA

2gukB

1lr0A

1rypL

1s2xA

1gu9C

2fd6U

1lslA

1rk8C

2hf1B

1xbwA

1ef1C

1u0sA

1ua8A

1q98A

1x9rA

2a07J

1je5A

2i3sB

2cw9A

2ouyB

1qr0A

2cy7A

2ovsA

1alu_

1qouB

2ftxA

1jhsA

1n67A

2ao9B

2qckA

2eboA

1jdhB

2gpeD

2nzhB

1nh2C

1v83A

2h18A

1b0nB

1fipA

1y9qA

1fleI

2i7fA

2gwlA

1mr8A

1d7bA

1dfnA

2b4yA

2dsyB

2nr5G

1zleA

2p0aA

1regX

2h0uA

1m0sA

1qzmA

1unnC

1gl2A

1gl2C

1gl2D

2o16B

2okuB

1sfp_

1ymqA

1xu1T

2hszA

1qqp4

2ht9B

1vkbA

1yiyA

2pmaA

2giaB

1o50A

1h1yA

1yb0B

2hv8F

1r75A

1ywmA

1uzxA

1gakA

2cxkA

1eb0A

1r9fA

1t0fC

1t15A

1qfoA

2is5C

2pjsA

1o6aA

2p7hA

1tz0A

1ze3H

1s7zA

2o8pA

2gjcA

2p8eA

1ry9A

2ewtA

1a34A

2hnfA

2gaxA

2hfnJ

1qkqA

1x8dA

1luzB

1n9pA

2ba2A

1a92D

1zw0B

1j22A

1nkpE

1wz3A

1fs1A

1s7iA

1r9wA

2j22A

1yodA

1lxjA

1dqeA

1g55A

1h7cA

1bb1A

1sc3B

1mwpA

1zn6A

2iiaA

1v54H

1v54I

1v54J

1v54K

1v54L

1xfkA

2ao3A

1no5A

1zuoA

2fi9A

2igpA

1dowB

2e1nA

2fygA

2p57A

2bfxD

1jyhA

1qwrA

2ikkA

2pstX

1q8dA

2co7A

1dusA

2iu1A

2uurA

1xiyA

1jidA

1kvdB

2c2iA

1ojhK

2cxhA

1umgA

2ip1A

2gwgA

2if6A

2ixdA

1vm0A

2i02A

1hxn_

1yacA

2acoA

2q03A

2omkA

1kp6A

1zvcA

1rgxC

2od5A

2giyA

1o5fL

2cjsC

1x7vA

2a6sB

2oyaA

2nyuA

1u9kA

1vi3A

2p3pA

1kw4A

2fpeG

1r77A

2f4qA

1pv5A

2d1gA

2ex4A

2gibB

1r0uA

1cv8_

2eteA

2fk9A

1kptA

2fe3A

3cla_

1vr8A

1jyaB

1pcfA

2i8gB

2p92A

1jmkC

1d0qA

1gxyA

1sf9A

2hp3A

1z7aA

1p3qQ

2g0cA

1q2hC

1tsfA

1ddwA

1qstA

1xzoB

1aqzB

1t2bA

1juvA

2buoA

2ds2D

1z0pA

1josA

2f5gA

2fckA

2hl7A

1ub4B

1zs4D

2py2A

2f37A

1zpsA

2gb7D

2o99D

2plwA

2jgbA

1k94A

1t1jA

1h6fB

2bopA

1h75A

1s5uG

1fltY

1x7iA

1mxiA

1nrjA

1zvbA

1nepA

1hz6B

1rh6B

2dtcA

1ecsA

1h03P

1n0wB

1vhh_

1dc1A

1j2lA

1pfvA

1vmbA

2hkvA

1orgA

1osyB

1oq1A

1j27A

1wdvA

1u5dB

2h5yA

1y63A

1yqhB

1ay7B

1urxA

1zlhB

2b8mA

1th7A

2f2bA

2fp7A

2a15A

2o1kA

1f60B

2bjfA

1dosA

2aj7B

1t0pB

2o30B

2isbA

1l8rA

1ufiD

1hfoA

1pq1B

1l6xB

1y66C

1pyoD

1dtdB

1ibyA

2oikC

1yo3A

1kcqA

2p09A

2bbaA

1sbxA

1w2yB

1x3kA

2ejnB

2p6yA

2o6fA

2aj6A

2i6vA

1xwwA

1d0dA

1htrP

2i3hB

1vjfA

1dj7A

1ro2A

1lj9B

1y4mA

1iujB

1wt6A

1kafE

1rz2A

1xppD

1g4yB

1vcc_

1tvgA

2b9dA

1it2A

1zv1A

2hbtA

1jhjA

2oy9A

1mavA

2d3dA

2d68B

1o26C

1gy7D

1t1vA

2cpgC

1j2jB

1wlzC

1zkeD

2pfiB

2bdrA

2ciuA

2d3mA

1mk0A

1t9iB

1euvA

1fcqA

1g8qB

1zngA

1nc5A

1v2bA

1xteA

1zmmB

2cxaA

2cxyA

1rkiB

2b7uA

2asfA

2aydA

1kqfC

1r5rA

1kicB

2dqaA

1b3aA

1p5zB

1g87A

2g2uB

2hlyA

1u69A

1y0uB

2a7mA

2qc0B

2iteA

1h2cA

2c6uA

2htdB

1hfeS

2gpiA

2nw0A

2jdcA

2okfA

2q3tA

2cdpD

2q9kA

2ia1B

1w0hA

2cmpA

1xs0C

2dxaA

1jovA

2a0b_

2hvfA

2j5sA

2ooaA

1eyhA

2afwA

2eixA

1kq1H

2askA

2f60K

1w4sA

1oqjB

1smbA

1na3A

2bemA

1vhwF

2avkA

1iv3A

2p17A

2a2kA

2o1rA

1hq1A

1whzA

1ypyB

1wrmA

2grcA

1zhvA

1nlqA

1v2xA

2czsB

1y5hB

1fl0A

1wmhA

1wmhB

2h7zA

1jl0B

1jekA

1jekB

1oi0D

1jr8B

1egwB

1d7pM

1wrvC

1qw2A

2p39A

2prxB

1h32A

1lmiA

1z3eA

1z3eB

1mtpB

1sj1A

2o6pB

2pa7B

1elkA

1dfmB

1dp7P

2h6fB

1zbfA

2iw1A

2dgeA

1r5lA

1qczA

1f7lA

1tp6A

2ds5B

2bayF

3ezmA

2q2fA

1opd_

2i5uA

1kgsA

1v5iB

1z3xA

2arcB

2cyjA

2fomA

2ibaA

2a35B

1t61D

1vyiA

1isuA

2orwB

2erbA

2ibdB

1uv4A

1ybiA

2imjD

2bkmA

2f62A

1dqzA

1yn9C

1uoyA

1lo7A

1x82A

1qwoA

1nf9A

1mg4A

2hx5A

2fnuB

2g1uA

1vqsB

2oplB

1zkpC

2j12A

1ocyA

1brt_

2ql8B

1gxnA

1bqcA

1my7B

2sns_

2q5cA

1z0nB

1ykuA

8abp_

1z1sA

1dzkA

1wpuA

1g6uA

3seb_

1upqA

2imsA

2olmA

2fr5C

2q3wA

1c5lL

1rkuA

1t6fA

2hzcA

1o82A

1gv9A

1g3p_

1vmgA

1nkoA

1xawA

1x2iA

1xyiA

1ybkC

2pq8A

1df4A

1idpA

1n7sB

1n7sD

1is3A

1xlqA

1hztA

1bgf_

2qfeA

1hlqA

1ji7C

2pq7A

1gvdA

1o9rD

2odiA

1i71A

2glzB

1zkkB

2hewF

1xd3C

1qqfA

1z67A

2f5tX

2cygA

1w1hD

1wl8A

2nn5A

2ckkA

1xbiA

1zdyA

2j8mB

2pr7A

2qjlA

2cjtC

1rjuV

2p7oB

2dplB

1m0kA

1pp0B

1pkhB

1nofA

2f46A

2bwqA

2p0nB

2pofA

2gu9B

1v30A

1jy2N

1jy2R

1jy2S

1ie9A

1es5A

1gp0A

2fq3A

2ohwA

1g8aA

1nycA

2ew0A

1gk7A

1pfbA

1y43A

1t7rA

1k7jA

1mnnA

2g7oA

1f8eA

1n13B

1uwwB

1ew4A

3vub_

1tzpB

1ecd_

1svfC

1svfD

2octB

1ypqB

1jl7A

2bu3B

2cb8B

2cxnA

2hoxB

2i51B

1q5yD

1ezgA

2fsqA

2cz2A

2j73B

1vjuB

1qopB

2axiA

2p8iC

1llfA

1k0mB

1q92A

2odkD

1q7lC

1q7lD

1qnrA

2jepB

1wb4A

1h2wA

1i1jB

2c3vB

2nuhA

1n8vB

1k55C

1ryqA

1s9uA

1hx0A

1f1eA

1jo0B

1wbeA

1wckA

2p8gA

2hd9A

1ijyA

1yn3A

1uzkA

1tzvA

1pb7A

1pz4A

1kufA

2i49A

2i24N

2nn8A

1vkkA

2gkpA

1gppA

2ossA

1sjwA

2jdaB

1gyxA

1oh4A

2iyvA

1eajB

2lisA

2qikA

2qe8B

1zgkA

1utg_

1vhuA

2ho2A

2uv4A

1plc_

1gpiA

1z0jB

2pmrA

1mkkB

1v9yA

2iblA

2q4nA

1kgdA

1vmhA

1jl1A

1f9vA

2ic2B

1v70A

2j9wB

2grrB

1ucdA

1xeoA

1zceA

1es9A

2agkA

1wcwA

2d5wA

2hxmA

2imfA

1hyoB

1yg9A

2d1sA

1o8xA

2nlvB

1useA

2gsoA

1cy5A

2izxB

2nr7A

2gzqA

2j6bA

2mhr_

1rutX

1tjyA

2g8oA

1flmA

1jcdB

2f69A

1gk9A

1gk9B

2e3bA

1wvfA

1nxmA

2j9cA

2ccvA

1lq9A

1w2lA

1dg6A

1o2dB

2oktA

1ka1A

1g61A

1qddA

2nqwA

1r29A

1dcs_

1oqvA

1o7qA

1pqhB

2dxuB

1rttA

1qksA

2pc1A

2cc6A

1gxuA

2fcwA

2fcwB

2gomB

1rdqE

2dy0B

2g3rA

1y6xA

2hy6G

2tpsB

2fvvA

2b82A

2c60A

1oohA

1jniA

2p2sA

2covI

1eaqB

1k3xA

1s3cA

2o37A

3chbD

2jcqA

1zmaA

2b3nB

1m4lA

1uscA

1mf7A

1i0vA

1w7cA

1kyfA

1mjuL

1mjuH

1d4oA

1e29A

1lb3A

1lf7A

1ryoA

2hlrA

1usmA

1v8hA

1ymtA

2pth_

1o7iB

1wn2A

2hhgA

1i24A

2cg7A

1oboA

2nrrA

2h8eA

1z2nX

1wxcA

1wxcB

1bx7_

1cseI

1tqwA

2a26B

2c1vA

1r5yA

1qu9A

1tu9A

1nz0D

1senA

2akfA

2flhD

2bmoA

2bmoB

2dlbA

1w6sB

1nwwB

1wc2A

1o08A

1ucrB

2f91B

1z0wA

1j98A

1knmA

1m15A

2iayA

1vr7B

2fkkA

1wm3A

2cs7C

2bk9A

1me4A

2fj8A

2frgP

1ifc_

1uwkB

1kq6A

2g7bA

1czpA

1c9oA

1h97A

1h4xA

1m1nA

1m1nB

2d8dB

2fhzA

2fhzB

2awkA

1k8uA

2bwfA

2oziB

1vfyA

1h4aX

2g8cO

2ciwA

1xmtA

1odmA

1vziA

1i8oA

1z70X

1e9gB

2nszA

2o9uX

2uuyB

1gwmA

1xt5A

1hdoA

2bzvA

1t3yA

1p6oB

1u07B

1jf8A

2eabB

1ra0A

1lu4A

2gzvA

1tukA

1k7cA

1sauA

2q3gB

2ofcA

1kt6A

1oh0B

2ii2A

1r0rI

1h4gB

1l3kA

1yfqA

1ys1X

1rg8A

1su8A

1uz3B

1t2dA

1bkrA

1ctj_

1f86A

2calA

1ls1A

2absA

1t8kA

1qv1A

1z2uA

2bhuA

1gmxA

1ql0A

2nwfA

2osxA

2aibA

1qj4A

2gqvA

1zl0B

1c5eA

2igd_

1n62C

1n62D

1k5nA

1k5nB

1nkd_

2carB

1qlwB

2h5oB

1w23B

1w66A

1uwcA

1jbeA

1ds1A

1xjuB

2ffyA

1sfsA

1pmhX

2dkoA

2dkoB

1kjqB

2hinB

1m9zA

2axwA

2czqA

1euwA

1m2dA

2d5mA

1lqtB

1kmvA

1rqwA

2hbwA

2c71A

2au7A

1fsgA

1yqsA

1psrA

1lwbA

1d5tA

1uowA

2gj3B

1i2tA

2bogX

2heuB

2anxA

1wuiS

1y93A

1kqpA

1cc8A

1cxqA

1rw1A

1i27A

1qtwA

1mfmA

1g2yB

1msoB

1y55X

1zk4A

1c7kA

1oaiA

2h3lB

1nqjB

2i4aA

1lkkA

1mn8D

1od3A

2iimA

2a6zA

2erl_

2jhfA

1tt8A

2pndA

1q6zA

2ggcA

1gkmA

1lniA

2cnqA

1ga6A

1o7jA

2cwsA

1eb6A

1jfbA

2chhA

2gkgA

1p1xB

1ug6A

1mwqB

1tqgA

1k4iA

1unqA

1ixh_

1xmkA

1m1qA

1zuuA

1f94A

1byi_

1c75A

2bf6A

1xg0B

1xg0D

1u2hA

1aho_

1k5cA

1ufyA

1nkiA

1wy3A

1kthA

1rtqA

1zzkA

1lugA

1mj5A

7a3hA

2fdn_

2bt9C

1kwfA

1vbwA

1ok0A

2g6fX

1l9lA

2fvyA

1n4wA

1iqzA

2nrlA

2pvbA

1j0pA

1et1A

1oewA

2bw4A

1vyrA

1g66A

1ix9A

1f9yA

1i1wA

1gweA

1v6pA

2ddxA

1muwA

1g6xA

2f01B

2fmaA

1pjxA

1x8qA

1mc2A

1m40A

2hs1A

2o7aA

1p9gA

2jfrA

1n55A

1nwzA

2h5cA

1w0nA

1pq7A

1iuaA

1x6zA

1gci_

2b97A

1r6jA

2dsxA

1us0A

1ucsA

1ejgA
