# Supplementary material for: Predictors of natively unfolded proteins: unanimous consensus score to detect a twilight zone between order and disorder in generic datasets
Source: BMC Bioinformatics. 2010 Apr 21;11:198. doi: 10.1186/1471-2105-11-198 (PMC2877690; doi:10.1186/1471-2105-11-198)
Supplement: Additional file 5 — DISPROT entries of unfolded proteins in set C. This file collects the DISPROT entries of the 81 natively unfolded proteins in set C. [file 1471-2105-11-198-S5.DOC]

**DISPROT entries of unfolded proteins in set C**

DP00001

DP00002

DP00005

DP00006

DP00008

DP00015

DP00016

DP00017

DP00022

DP00024

DP00027

DP00028

DP00038

DP00039

DP00040

DP00041

DP00042

DP00047

DP00048

DP00057

DP00058

DP00068

DP00069

DP00070

DP00075

DP00112

DP00116

DP00122

DP00124

DP00126

DP00128

DP00132

DP00136

DP00139

DP00140

DP00143

DP00145

DP00146

DP00147

DP00148

DP00158

DP00163

DP00170

DP00174

DP00186

DP00188

DP00192

DP00193

DP00198

DP00199

DP00201

DP00205

DP00207

DP00214

DP00216

DP00219

DP00222

DP00227

DP00232

DP00242

DP00253

DP00281

DP00287

DP00288

DP00303

DP00325

DP00328

DP00330

DP00332

DP00333

DP00347

DP00357

DP00359

DP00367

DP00372

DP00387

DP00421

DP00441

DP00465

DP00510

DP00521
